# ALMS1 and Alström syndrome: a recessive form of metabolic, neurosensory and cardiac deficits

**DOI:** 10.1007/s00109-018-1714-x

**Published:** 2018-11-12

**Authors:** Tom Hearn

**Affiliations:** Institute of Life Science, Swansea University Medical School, Singleton Park, Swansea, SA2 8PP UK

**Keywords:** Actinin, ALMS motif, Bardet-Biedl syndrome, C10orf90, CART complex, Centriole, Centrosome, CEP250, CEP295, C-Nap1, Cilia, Ciliopathy, Diabetes, Endosomal recycling, Fibrosis, GLUT4, Insulin resistance, KIAA1731, Mitogenic cardiomyopathy, Notch, Obesity, Pericentrin, RFX, Rootlet

## Abstract

Alström syndrome (AS) is characterised by metabolic deficits, retinal dystrophy, sensorineural hearing loss, dilated cardiomyopathy and multi-organ fibrosis. Elucidating the function of the mutated gene, *ALMS1*, is critical for the development of specific treatments and may uncover pathways relevant to a range of other disorders including common forms of obesity and type 2 diabetes. Interest in *ALMS1* is heightened by the recent discovery of its involvement in neonatal cardiomyocyte cell cycle arrest, a process with potential relevance to regenerative medicine. *ALMS1* encodes a ~ 0.5 megadalton protein that localises to the base of centrioles. Some studies have suggested a role for this protein in maintaining centriole-nucleated sensory organelles termed primary cilia, and AS is now considered to belong to the growing class of human genetic disorders linked to ciliary dysfunction (ciliopathies). However, mechanistic details are lacking, and recent studies have implicated ALMS1 in several processes including endosomal trafficking, actin organisation, maintenance of centrosome cohesion and transcription. In line with a more complex picture, multiple isoforms of the protein likely exist and non-centrosomal sites of localisation have been reported. This review outlines the evidence for both ciliary and extra-ciliary functions of ALMS1.

## Introduction

Alström syndrome (AS) is a rare, recessively inherited disorder that affects numerous organ systems [1, 2]. Cardinal features include childhood obesity, insulin resistance, cone-rod retinal dystrophy and sensorineural hearing loss (SNHL). Type 2 diabetes mellitus and hypertriglyceridemia typically develop in childhood or adolescence [2–4]. Dilated cardiomyopathy (DCM) may arise in infancy, then often resolve or at least abate within 3 years. However, it may recur, or manifest for the first time, in adolescence or adulthood [2, 3, 4, 7]. Other common features include hepatic, renal and pulmonary dysfunction, chronic otitis media in childhood, abnormal spinal curvature (scoliosis, kyphosis or lordosis) arising in adolescence, short stature in adulthood, gastrointestinal disturbances and neurological disturbances such as absence seizures [3–6]. Fibrosis of multiple organs is a common finding [4].

Understanding the molecular basis of AS is critical for the development of specific therapies. It may also provide new insights into the molecular pathways that go awry in common forms of conditions such as obesity and diabetes, which represent major public health concerns [8, 9].

The prevalence of AS is estimated at 1–9 cases per million individuals, with approximately 950 reported cases [10]. It is caused by mutations in *ALMS1*, a widely expressed 23-exon gene located on chromosome 2p13 [11, 12]. Over 200 different AS-causing variants have been identified, of which the vast majority are nonsense or frameshift mutations [13, 14]. Almost half occur in exon 8 [14], commensurate with the large size of this exon (6.1 kb, or 49% of the coding sequence). There is little evidence of genotype-phenotype correlation, suggesting that the phenotype is modified by unknown genetic and/or environmental factors [14].

Intriguingly, biallelic mutations in *ALMS1* also cause mitogenic cardiomyopathy, an ultra-rare form of DCM characterised by delayed cell cycle arrest of neonatal cardiomyocytes [15, 16]. This condition is fatal in the first months of life, before other clinical features of AS normally manifest. The few mutations reported so far resemble those commonly found in AS, being located in the three largest exons (exons 8, 10 and 16) and predicted to cause premature termination of translation. Alms1 also appears to be required for timely postnatal cell cycle arrest of cardiomyocytes in mice [15]. Thus, mitogenic cardiomyopathy is most likely an extreme form of the infantile DCM often observed in AS [15, 16]. Mechanisms regulating cardiomyocyte cell cycle arrest are of great interest partly because reversing this process could provide a way to stimulate cardiac regeneration after injury [17].

**Fig. 1:**
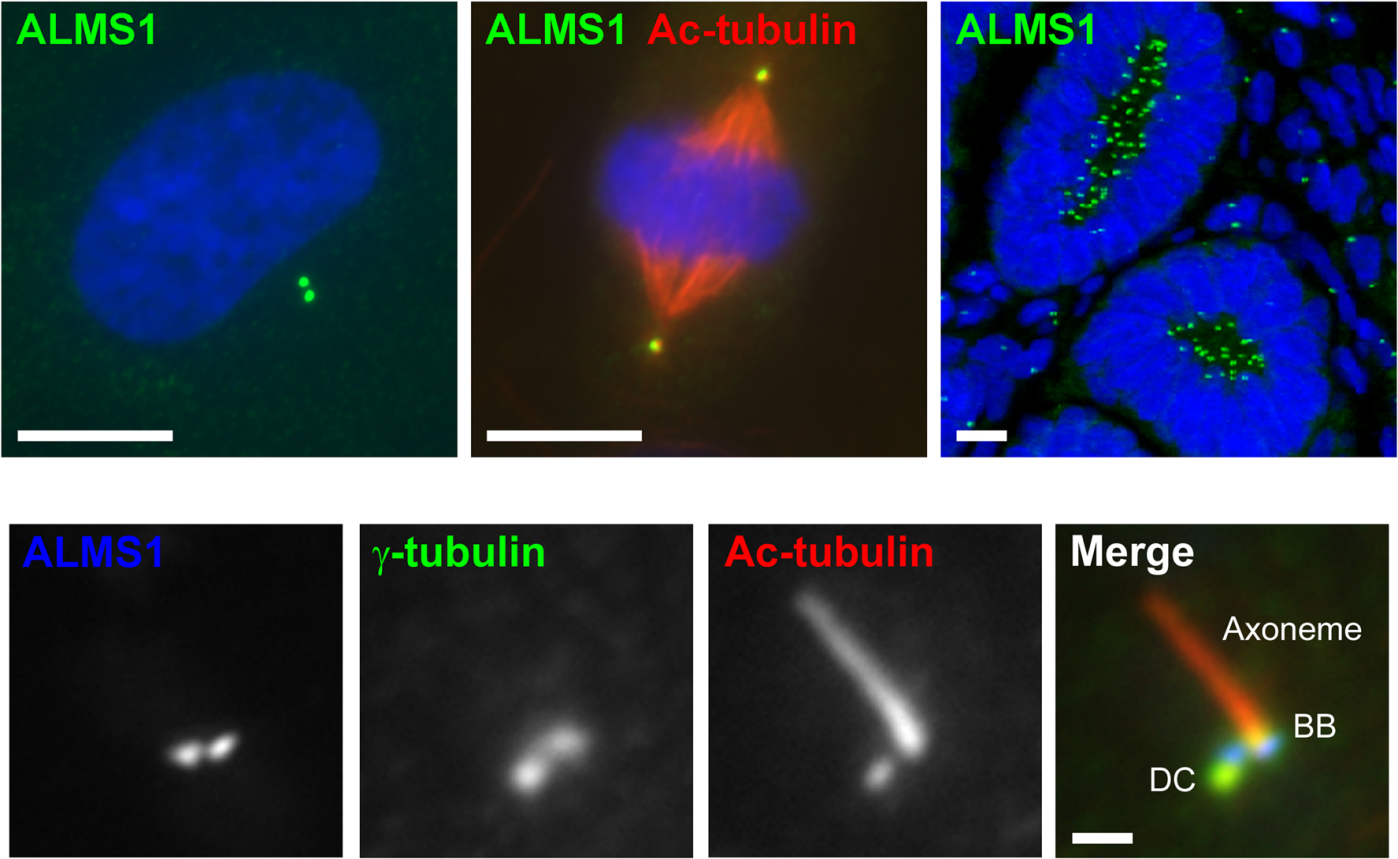
ALMS1 is a centrosomal protein that localises specifically to the proximal end of centrioles/basal bodies. Top (l-r): immunofluorescence microscopy images showing centrosomal staining pattern of ALMS1 in hTERT-RPE1 cells in interphase and mitosis, and in fetal human kidney. The mitotic hTERT-RPE1 cell was co-stained with an antibody to acetylated α-tubulin (Ac-tubulin), which marks centrioles and spindle microtubules. DNA was stained with DAPI (blue). Scale bars, 10 μm. Bottom: ciliated hTERT-RPE1 cell stained with antibodies to ALMS1, the PCM component γ-tubulin and the centriole/axoneme component Ac-tubulin. Localisation of ALMS1 to centriole/basal body proximal ends has been confirmed by super-resolution microscopy [21]. *DC* daughter centriole, *BB* basal body. Scale bar, 1 μm

**Fig. 2:**
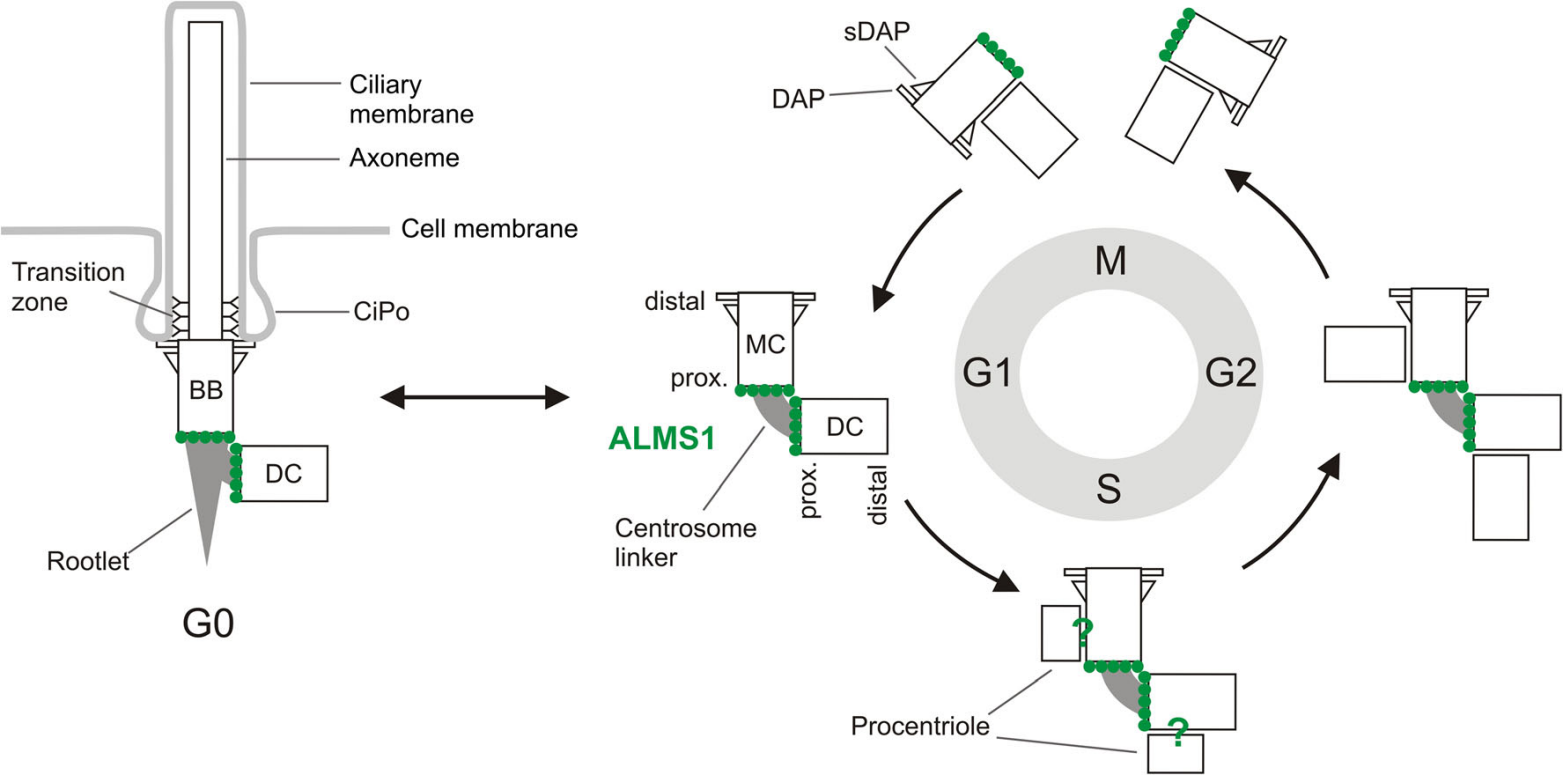
Primary cilia and the centrosome cycle. In G1 phase of the cell cycle the centrosome contains one mother and one daughter centriole (MC, DC), the MC being distinguished by appendages at its distal end. In S and G2 phases, one procentriole assembles orthogonally to each pre-existing centriole [20]. ALMS1 (green circles) localises to the proximal ends of the MC and DC, from where the centrosome linker extends; it is not known if ALMS1 localises to procentrioles (green question marks). The centrosome linker is dissolved before mitotic entry, facilitating bipolar spindle formation, and reassembles at the end of mitosis when the newly built centriole disengages from its parent’s side. The pre-existing DC acquires appendages in late G2 or M phase and functions as an MC in the following cell cycle [20]. Upon cell cycle exit (G0), most cell types generate a primary cilium. The MC alone is competent to form a cilium, and in this role is termed a basal body (BB). Cilia are typically resorbed upon cell cycle re-entry. However, in some cell types/contexts, they may persist, or re-assemble in interphase, before disassembling prior to mitosis (not shown) [183]. *CiPo* ciliary pocket, *DAP* distal appendages, *sDAP* subdistal appendages

The ALMS1 protein is a component of the centrosome [18, 19], a microtubule (MT)-nucleating organelle comprising two centrioles (termed mother and daughter) and a dynamic assembly of proteins termed pericentriolar material (PCM). Centrioles are evolutionarily conserved cylindrical structures composed of nine triplet MTs, approximately 500 nm in length [20]. Notably, ALMS1 localises specifically to the base, or proximal end, of these structures [21] (Figs. 1 and 2).

Centrioles duplicate once per cell cycle, beginning at the G1/S transition, and the two resulting centrosomes go on to form the poles of the mitotic spindle [20] (Fig. 2). In interphase, and in some terminally differentiated cell types, the centrosome anchors a radial array of MTs that forms the basis of an intracellular transport system and influences cell shape, polarity and motility [22, 23]. In addition to its classical roles in MT organisation, the centrosome has recently been reported to nucleate actin filaments, facilitate endocytic recycling and direct multiple events at the immunological synapse [24–26]. It also serves as a hub for components of the cell cycle regulatory machinery and proteasome, and has been linked to the DNA damage response (DDR) [27–29]. Of note, mutations in genes encoding the centrosomal proteins pericentrin (PCNT) and POC1A are associated with severe insulin resistance [30, 31], while disruption of the centrosomal protein CEP19 causes morbid obesity [32]. Mutations in several centriole biogenesis genes cause forms of microcephaly [20].

Centrioles are vital for the assembly of cilia, hair-like projections that are either motile, as in multiciliated epithelial cells (MCCs), or non-motile. Most quiescent/terminally differentiated (G0) human cells possess a single non-motile (primary) cilium, nucleated by the mother centriole of the centrosome (termed a basal body when ciliated) (Fig. 2). The core, or axoneme, of primary cilia consists of nine doublet MTs and is enclosed in a lipid bilayer that is continuous with the plasma membrane [33]. In many cell types, the periciliary membrane forms an invagination called the ciliary pocket, which is a site of endocytosis and possibly a docking site for Golgi-derived vesicles [34]. Protein synthesis does not occur within cilia and therefore all components must be transported from the cell body. A continuously running MT motor-based system termed intraflagellar transport (IFT) shuttles cargo molecules from the base to the tip of the cilium and vice versa [33]. Importantly, the protein and lipid composition of the ciliary compartment is controlled by a ‘gate’ at the ciliary base, part of which is the transition zone (TZ), defined by Y-shaped links extending from the axoneme to the ciliary membrane [35] (Fig. 2).

Notably, the light-sensing outer segment (OS) of vertebrate photoreceptors is a highly specialised form of primary cilium [36]. The OS is packed with stacks of opsin-containing membrane disks that are shed from the distal end and renewed at the base. This requires high-volume trafficking of OS components from their site of synthesis in the inner segment (IS) through a narrow structure called the connecting cilium (CC), which resembles an extended TZ and is nucleated by a basal body in the distal IS [35, 36]. Another specialised type of primary cilium is present on hair cells in the developing organ of Corti. This ‘kinocilium’ and/or its basal body help to choreograph the formation of mechanically sensitive bundles of stereocilia (actin-based structures unrelated to primary cilia), which are central to hearing, on the apical cell surface [37, 38].

Interest in primary cilia has widened dramatically in recent years due to accumulating evidence of their importance for normal development, tissue homeostasis and energy balance [39–41]. They appear to sense and process a variety of molecular cues, helping cells throughout the body to respond to changes in the extracellular environment. For example, vertebrate cells require primary cilia to respond to Hedgehog (Hh) family ligands, which play crucial roles in development and stem cell maintenance [42]. The signalling process involves dynamic localisation of effector transcription factors and other pathway components to the ciliary compartment [42]. In growth-arrested cells, platelet-derived growth factor receptor α (PDGFRα) and insulin-like growth factor 1 receptor (IGF-1R) localise to the ciliary membrane, where they trigger downstream signalling upon ligand-binding, leading to responses such as cell cycle re-entry [43–45]. Primary cilia are involved in numerous other signalling pathways, including the Wnt/PCP (planar cell polarity), Notch and TGF-β pathways, and may thus serve to integrate multiple signals to ensure an appropriate cellular response [39, 46–48]. The primary cilia of some cells, such as renal tubule epithelial cells, are thought to sense fluid flow via mechanosensitive calcium channel complexes within the ciliary membrane [49]. However, this aspect of cilia function has been questioned by recent data [49, 50].

While cilia-mediated signalling can influence the cell cycle, ciliation status is in turn controlled by cell cycle kinases [51]. Moreover, ciliogenesis and/or cilium length are modulated by inputs from other processes such as autophagy, actin cytoskeleton organisation and ciliary signalling itself [51].

Dozens of genetic disorders have now been linked to cilia dysfunction [52]. Clinical features common to several of these so-called ciliopathies include renal cysts, brain abnormalities, polydactyly and retinal dystrophy [53–55]. The products of many of the mutated genes have been shown to work together in complexes or functional modules, helping to explain both the genetic heterogeneity of several ciliopathies and the many examples of genetic overlap [52–54]. ALMS1 loss-of-function has been linked to defects in primary cilium formation, positioning and maintenance, leading to AS being classified as a ciliopathy [56–59]. However, the precise role of the ALMS1 protein remains uncertain and no clear links have been established with other ciliopathy proteins.

The phenotype of AS resembles that of the genetically heterogeneous ciliopathy Bardet-Biedl syndrome (BBS), which includes childhood obesity, retinal dystrophy, SNHL and susceptibility to type 2 diabetes [9, 60]. The functions of many BBS proteins converge at an evolutionarily conserved protein complex termed the BBSome [61, 62], to which various functions have been ascribed including promotion of ciliary membrane biogenesis, trafficking of ciliary membrane proteins and regulation of IFT particle assembly [61, 63–65]. BBS proteins also appear to have extraciliary functions, for example mediating trafficking of the insulin and leptin receptors to the plasma membrane [66, 67].

AS also bears similarity to the phenotype of mice with a mutation in the *Tub* gene, characterised by obesity, insulin resistance, retinal degeneration and SNHL [68, 69]. The Tubby protein is implicated in trafficking G protein-coupled receptors (GPCRs) and transient receptor potential (TRP) channels to neuronal cilia [70, 71], and may also act as a transcription factor [72].

While ALMS1 is known to localise to centrioles/basal bodies [19, 21, 56, 57], localisation to non-centrosomal sites has also been reported and recent studies have implicated the protein in several cellular processes including endosomal trafficking, actin organisation and transcription [73–77]. On broader scales, ALMS1 has been implicated in neuronal migration, maintenance of cellular quiescence, adipogenesis, spermatogenesis, maintenance of pancreatic β cell mass, adaptive thermogenesis and, as noted above, cell cycle arrest of cardiomyocytes [15, 16, 57, 78–82].

This review focuses on the quest to understand AS at the molecular and cell biological levels. In addition to results from mouse model- and cell-based functional studies, recent insights into *ALMS1* gene regulation and the interaction network of the encoded protein are highlighted.

## The ALMS1 protein

**Fig. 3:**
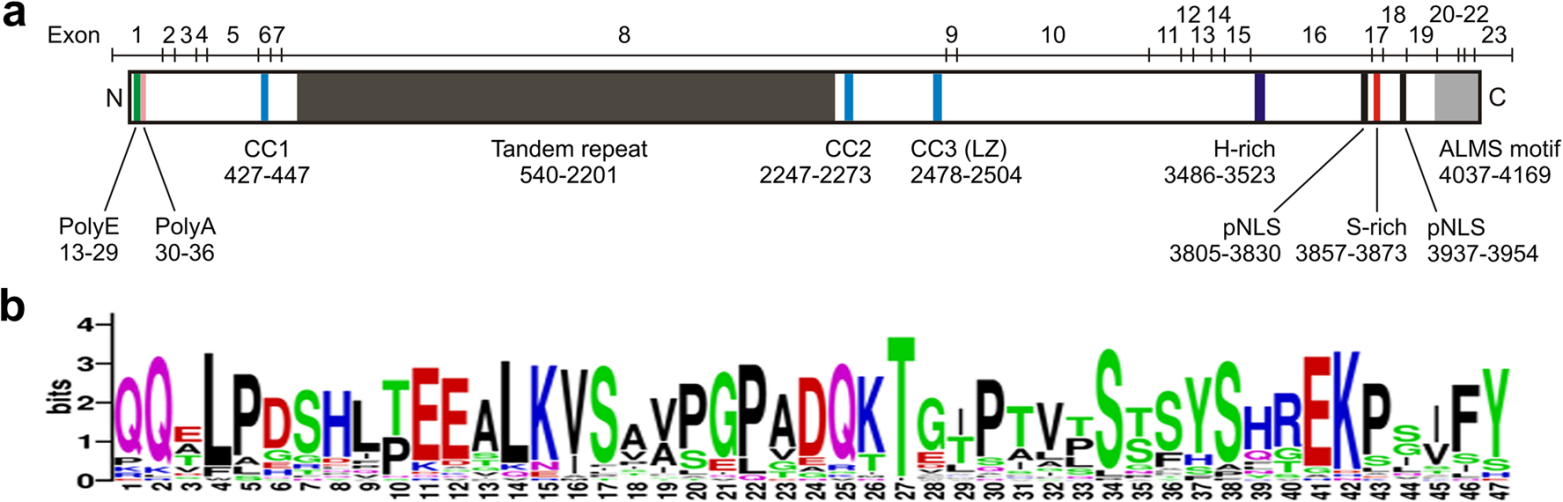
Sequence features of ALMS1. **a** Representation of the primary structure showing recognised features in relation to exon boundaries. The N-terminal polyglutamate (PolyE) tract is polymorphic [12] and is followed by seven alanine residues. Short lengths of the polyE tract have been associated with an increased risk of early-onset myocardial infarction in the general population [184]. *CC* predicted coiled-coil domain, *LZ* leucine zipper motif, *pNLS* potential nuclear localisation signal. Numbers below each feature refer to amino acid positions [11, 12, 21]. **b** Consensus sequence of the tandem repeat. The repeats are relatively rich in serine and proline residues and have little secondary structure-forming potential. Consensus generated using WebLogo [185], based on an alignment with seven gaps removed to facilitate display

ALMS1 is a large (> 4000 residue) protein that lacks known catalytic domains [11, 12]. It has several sequence features of unknown function, including a large tandem repeat domain (TRD), three short predicted coiled-coil domains and a stretch of ~ 130 residues at the C-terminus dubbed the ALMS motif (Fig. 3). The ALMS motif is the only region that shares obvious sequence similarity with other human proteins. It was defined on the basis of similarity to mammalian orthologues of C10orf90/FATS (fragile site-associated tumour suppressor) [11], reportedly an E2-independent ubiquitin ligase which stabilises p53 in response to DNA damage [83] and may localise to the centrosome and actin cytoskeleton [21]. A more divergent ALMS motif was subsequently identified at the C-terminus of CEP295/KIAA1731 [21], a large protein implicated in centriole assembly and maintenance [21, 84–87]. Notably, the CEP295 ALMS motif appears to bind MTs [84]. Deletion analysis of ALMS1 suggests that its ALMS motif may contribute to centrosome-targeting [21]. However, the motif is not essential for centrosomal localisation of either ALMS1 or CEP295 [21, 84, 85].

Orthologues of ALMS1 are readily detectable in chordates, although most of the N-terminal half of the protein, which mainly comprises the TRD, appears to be specific to mammals [21; unpublished observation]. The proteomes of many ciliated eukaryotes, including unicellular organisms such as Tetrahymena and Paramecium, contain predicted proteins with C-terminal ALMS motif-like sequences, suggesting that the motif has an ancient, conserved function relating to centrioles or cilia [21].

Experimentally defined sites of post-translational modification in ALMS1 include a cluster of ubiquitination sites around the third predicted coiled-coil domain [88] and a DNA damage-regulated phosphorylation site (S2781) [89] (data accessed via PhosphoSitePlus [90]). Regarding the latter, it is notable that a genome-wide RNAi screen identified *ALMS1* as one of ~ 800 genes required for resistance to ionising radiation in U2OS cells [91]. Other ciliopathy-associated proteins have been linked to DDR signalling [92] but the potential role of ALMS1 in this process remains to be investigated.

## Regulation of *ALMS1* gene expression

*ALMS1* is widely expressed [11, 12, 19, 93] but its level of expression is context-dependent. For example, *Alms1* mRNA declines during in vitro differentiation of 3T3-L1 preadipocytes [79, 94], possibly related to the observation that mature adipocytes lack primary cilia [95]. Conversely, it increases during terminal differentiation of neonatal mouse cardiomyocytes [15] and in cell culture conditions that promote cell cycle exit and primary cilium formation (i.e. serum-starvation or confluency) [57, 96, 97]. It also increases during differentiation of MCCs in vitro [98, 99], possibly reflecting a requirement for the protein at the hundreds of basal bodies assembled in these cells.

The *ALMS1* promoter contains an evolutionarily conserved consensus binding site for ciliogenic RFX (regulatory factor X) transcription factors, three of which (RFX1, RFX2 and RFX3) have been implicated in activating *ALMS1* transcription in response to serum-starvation [96]. Involvement of RFX proteins is supported by ChIP-Seq data from other studies, for example for murine Rfx1 and Rfx3 in neural progenitors and Min6 insulinoma cells, respectively [100, 101] (data accessed via the Cistrome Data Browser [102]). Vertebrate RFX2 and RFX3 have well-established roles in regulating ciliary genes in various tissues [103], while RFX1 has recently been reported to stimulate transcription of key ciliogenic genes downstream of leptin in hypothalamic neurons and to regulate other ciliary genes [104, 105]. However, it should be noted that RFX1 and RFX3 also regulate non-ciliary genes [100, 103, 106].

In *Xenopus* differentiating MCCs, *Alms1* transcription appears to be upregulated by a protein complex containing Multicilin and the DNA-binding transcription factor E2f4 [99]. Many of the genes targeted by this complex in MCCs encode factors that, in proliferating cells, are known to be involved in centriole biogenesis [99].

## Protein-protein interactions and proximity interactions

Numerous candidate ALMS1-interacting proteins have been reported. Members of the α-actinin family of actin-binding proteins were identified in yeast two-hybrid (Y2H) screens using the C-terminal region of murine Alms1 as bait, and co-immunoprecipitation data supported an interaction with α-actinin-4 (Actn4) in mouse kidney [76]. ACTN4 and another protein identified in the same Y2H screen (myosin Vb) are components of the cytoskeleton-associated recycling or transport (CART) complex, involved in endosomal recycling [107]. Myo5b also interacts with a key regulator of ‘slow’ recycling pathways, Rab11 [108], and shares the ability of Myo5a to interact with the ciliopathy protein RPGRIP1L [109]. α-Actinins and another protein identified in the same Y2H screen, Rab-interacting lysosomal protein-like 1 (Rilpl1), have also been implicated in ciliary roles [110, 111].

An ongoing human interactome study based on affinity purification coupled with mass spectrometry (AP-MS) [112, 113] has identified 18 potential ALMS1 interactors including the BBSome subunit BBS7; AVIL (advillin), which severs actin filaments and influences ciliogenesis [114]; RABL2A, the mouse orthologue of which binds CEP19 and IFT proteins [115–117]; cell division cycle 16 (CDC16), a component of the anaphase-promoting complex; dynamin 3 (DNM3), involved in membrane fission [118]; TFDP3, a dimerization partner of E2F transcription factors [119]; and dysbindin/dystrobrevin binding protein 1 (DTNBP1), a subunit of the BLOC-1 complex that is involved in recycling endosome formation and GPCR trafficking in the lysosomal pathway, and is required for trafficking of polycystin-2 to cilia [120–122]. Another global AP-MS study identified an interaction with VCIP135/valosin-containing protein-interacting protein 1 (VCPIP1) [123], a deubiquitinating enzyme which functions in membrane fusion [124, 125].

Other AP-MS studies have identified ALMS1 as a possible binding partner of the RNA polymerase II (RNAPII) subunit RPB1 [77]; the serine-arginine protein kinase SRPK2 [126]; GPCR-associated sorting protein 2 (GPRASP2), implicated in ciliary translocation of Smoothened, a key component of the Hh signalling pathway [127, 128]; and CEP192, a protein involved in centrosome biogenesis [129]. Consistent with the latter finding, ALMS1 was labelled by a CEP192-biotin ligase fusion (bait) in a proximity-dependent biotinylation (BioID) study [130]. However, because ALMS1 was labelled by baits representing several centriole duplication factors (PLK4, CEP152, CPAP, CEP63 and KIAA0753), it was suggested that at least in some cases this may reflect a dispersed localisation or high mobility within the centrosome [130]. In a BioID study focusing on centrioles, cilia and centriolar satellites, centriole assembly factors including CEP152, CPAP, CEP135 and SASS6 were among 11 baits producing high confidence proximity interactions with ALMS1 [131]. Collectively, these data may indicate that ALMS1 is present at the proximal ends of nascent procentrioles. However, the timing of ALMS1’s recruitment to newly forming centrioles is unknown, and it remains to be determined if the BioID results reflect physical interactions.

BioID has also identified ALMS1 as a potential interactor/substrate of the E3 ubiquitin ligase SCF^β-TrCP1^ [132], and of separase [133], a protease that mediates separation of sister chromatids, and of newborn centrioles from their parents, in mitosis [134]. Another recent BioID study suggests links to proteins involved in the Hippo signalling pathway (LATS2 and AMOT; [135]). An updated list of reported physical and proximity interactions is available online at thebiogrid.org [136].

## ALMS1 function

### Cilium formation/maintenance

The notion that ALMS1 has a ciliary role probably first arose when BBS was linked to basal body/primary cilium dysfunction [137]. As outlined below, supporting evidence has emerged from analyses of ALMS1-deficient mice and ALMS1-depleted cultured cells, although not to date from analyses of AS patient fibroblasts.

#### Alms1^GT/GT^ mice

**Fig. 4:**
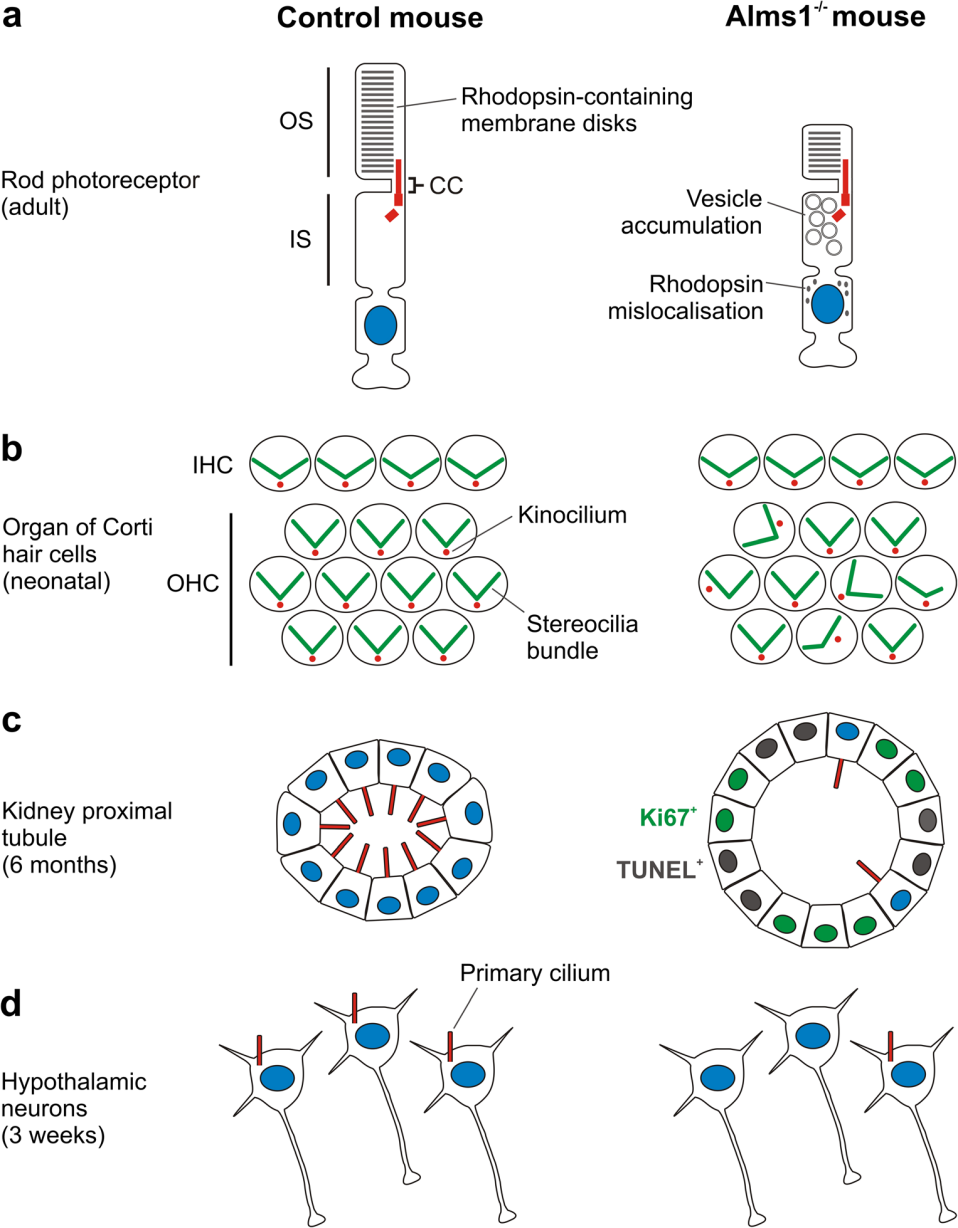
Selected cellular phenotypes observed in mouse models of AS. **a** Large membrane-bound vesicles accumulate in the inner segment (IS) of adult *Alms1*^GT/GT^ rod photoreceptors. Immunofluorescence data indicate shortening of the inner and outer and segments (OS) at 24 weeks of age and mislocalisation of rhodopsin at 7 and 24 weeks of age [93]. Similar mislocalisation of rhodopsin occurs in *Alms1*^*L2131X/L2131X*^ retina [57]. It is not known if mislocalised rhodopsin is vesicle-associated. **b** In neonatal *Alms1*^GT/GT^ mice, some outer hair cells (OHC) in the organ of Corti display misoriented and/or misshapen bundles of stereocilia (actin-based structures) and mislocalisation of kinocilia (a type of primary cilium), indicating impaired cilium-dependent planar cell polarity. Inner hair cell (IHC) bundles appear unaffected [56]. **c** Representation of kidney proximal tubule epithelium in six month-old WT and *Alms1*^*L2131X/L2131X*^ mice, based on data indicating tubule dilation and loss of primary cilia coupled with increased proliferation (Ki67 staining) and apoptosis (TUNEL staining) in the mutant [57]. **d** Loss of hypothalamic neuronal primary cilia occurs by 3 weeks of age in *Alms1*^*foz/foz*^ mice [58]

Mice with a gene trap in intron 13 (*Alms1*^GT/GT^ mice) appear to form cilia normally, based on electron microscopy analysis of renal collecting ducts, photoreceptors and airway epithelium [93]. However, within photoreceptors a fraction of rhodopsin mislocalises to the outer nuclear layer (ONL) and vesicles accumulate in the IS, suggesting a defect in the tethering, docking or fusion of rhodopsin carrier vesicles at the base of the connecting cilium [93] (Fig. 4a). Photoreceptor degeneration is evident by the age of 24 weeks in these mice.

In the developing organ of Corti in *Alms1*^GT/GT^ mice, the kinocilium of outer hair cells (OHC) is often mis-positioned and some stereociliary bundles display defects in shape and orientation [56] (Fig. 4b). These abnormalities, which resemble those seen in mouse models of BBS [138], implicate ALMS1 in cilium-dependent PCP signalling [56]. The kinocilium appears to be required for normal bundle shape and orientation in individual OHCs, but not for polarisation of core PCP proteins [38]. Only ~ 20% of OHC bundles are affected in *Alms1*^GT/GT^ mice, suggesting that Alms1 dysfunction renders this cell type susceptible to a stochastic event during development [56]. Progressive loss of OHCs also occurs and appears to be the main cause of hearing loss in these mice, although it is unclear if this is directly related to the observed bundle abnormalities [56].

#### Alms1^L2131X/L2131X^ mice

Mice with an *N*-ethyl-*N*-nitrosourea (ENU)-induced nonsense mutation in exon 10 (*Alms1*^*L2131X/L2131X*^ mice) exhibit loss of renal primary cilia by 6 months of age [57]. Notably, only certain proximal tubules are affected, suggesting that an additional, somatic, event may be required [57]. Cilia-loss correlates with increased proliferation and apoptosis of tubule epithelial cells [57] but whether it triggers these changes remains unknown (Fig. 4c). Rhodopsin mislocalises to the ONL in some photoreceptors of *Alms1*^*L2131X/L2131X*^ mice, similar to *Alms1*^GT/GT^ mice. Also of note, sperm flagella, which are structurally similar to cilia, are reduced in number and length compared to wild-type [57].

#### Alms1^foz/foz^ mice

Mice with a truncating mutation in exon 8 (*Alms1*^*foz/foz*^ mice) have also been reported to exhibit age-dependent loss of primary cilia, in this case on hypothalamic neurons [58]. The loss begins between 5 days and 3 weeks of age, based on staining for AC3 (adenylate cyclase type 3), and seems to occur rapidly because the remaining cilia are not significantly shorter than those of control mice (Fig. 4d). Loss of cilia was supported by staining for three additional ciliary proteins (Sstr3, Mchr1 and Arl13b). Because the markers used are not core structural components of cilia, it is formally possible that their trafficking to cilia is blocked in subsets of *Alms1*^*foz/foz*^ neurons. However, on the reasonable assumption that cilia are lost, the detection of Sstr3 and Mhcr1 in remaining cilia [58] is notable because ciliary localisation of these GPCRs is abrogated in *Bbs* and *tubby* mutant mice [71, 139].

Hypothalamic neuronal primary cilia are implicated in regulating satiety responses (via factors including Mchr1 and AC3) [139–141] and *Alms1*^*foz/foz*^ mice are reportedly hyperphagic before becoming obese [82]. Thus, resorption or degeneration of these cilia may contribute to the development of obesity in these mice [58]. Although *Alms1*^GT/GT^ and *Alms1*^*L2131X/L2131X*^ mice also become obese [57, 93], preceding hyperphagia has not been documented and it is not known if loss of hypothalamic primary cilia occurs. Another notable feature of *Alms1*^*foz/foz*^ mice is the degeneration of germ cells during spermatogenesis, particularly at the round-to-elongating stage during which flagella assembly occurs, although the assembly process is completed in some cells [82].

In summary, the initial formation of primary cilia appears normal in all three mouse models reported to date, consistent with their lack of obvious developmental phenotypes. However, age-dependent loss of primary cilia has been observed in *Alms1*^*L2131X/L2131X*^ and *Alms1*^*foz/foz*^ mice. Cilia-related deficits in a third model (*Alms1*^GT/GT^ mice) include mislocalisation of rhodopsin and mispositioning of OHC kinocilia. Whether differences in the occurrence/timing of cilia-loss seen in these studies reflect the nature of the mutations, the focus on different cell types, or other factors, remains uncertain.

#### AS patient fibroblasts and ALMS1 siRNA-treated cells

The formation of primary cilia seems to be unaffected in AS patient fibroblasts in vitro [19, 76, 142]. Hh and PDGFA signalling, both of which act through the cilium, also appear to be normal [142]. In stark contrast, transient RNAi-mediated depletion of ALMS1 has been reported to cause severe defects in ciliary structure. Ball-like staining of the ciliary axoneme marker acetylated tubulin was apparent in Alms1-depleted mouse inner medullary collecting duct (IMCD3) cells, using two different siRNAs [57]. These malformed cilia were also functionally defective, based on measurement of intracellular calcium in response to fluid flow. A cDNA construct encoding the N-terminal 40% of Alms1 was reported to rescue cilium formation, although the effect was not quantified and the subcellular localisation of this N-terminal fragment was not reported [57]. Knockdown of ALMS1 in human telomerase-immortalised retinal pigmented epithelium (hTERT-RPE1) cells has similarly been reported to result in the appearance of morphologically abnormal, stunted cilia [59], although whether the two siRNAs tested produced exactly the same effect is unclear.

Notably, Alms1 immunofluorescence is clearly visible at centrosomes in *Alms1*^*L2131X/L2131X*^ and *Alms1*^*GT/GT*^ mice, but not Alms1-depleted IMCD3 cells, using an N-terminal antibody [56, 57]. Moreover, Alms1 siRNA treatment has been reported to inhibit ciliogenesis in *Alms1*^*L2131X/L2131X*^ embryonic fibroblasts [57]. These findings suggest that RNAi suppresses ALMS1 expression more comprehensively than truncating mutations, at least in murine cells, offering a possible explanation for the striking effects on cilium morphology [57]. If this is the case, then true null mutations in *ALMS1* would likely disrupt ciliogenesis (assuming lack of adaptive compensation), leading to severe developmental defects and possibly embryonic lethality.

On the other hand, it could be argued that additional data are needed to confirm that the catastrophic ciliary defects seen in siRNA-treated cells are due to depletion of ALMS1. It is notable that Alms1 immunostaining was not detected in *Alms1*^*foz/foz*^ neurons [58], nor in fibroblasts from 14 out of 16 AS patients with biallelic nonsense/frameshift mutations [142], implying the absence of residual protein function. Nevertheless, expression of ALMS1 isoforms lacking the antibody epitopes used for detection cannot be excluded in these cases.

### Maintenance of centrosome cohesion and composition

**Fig. 5:**
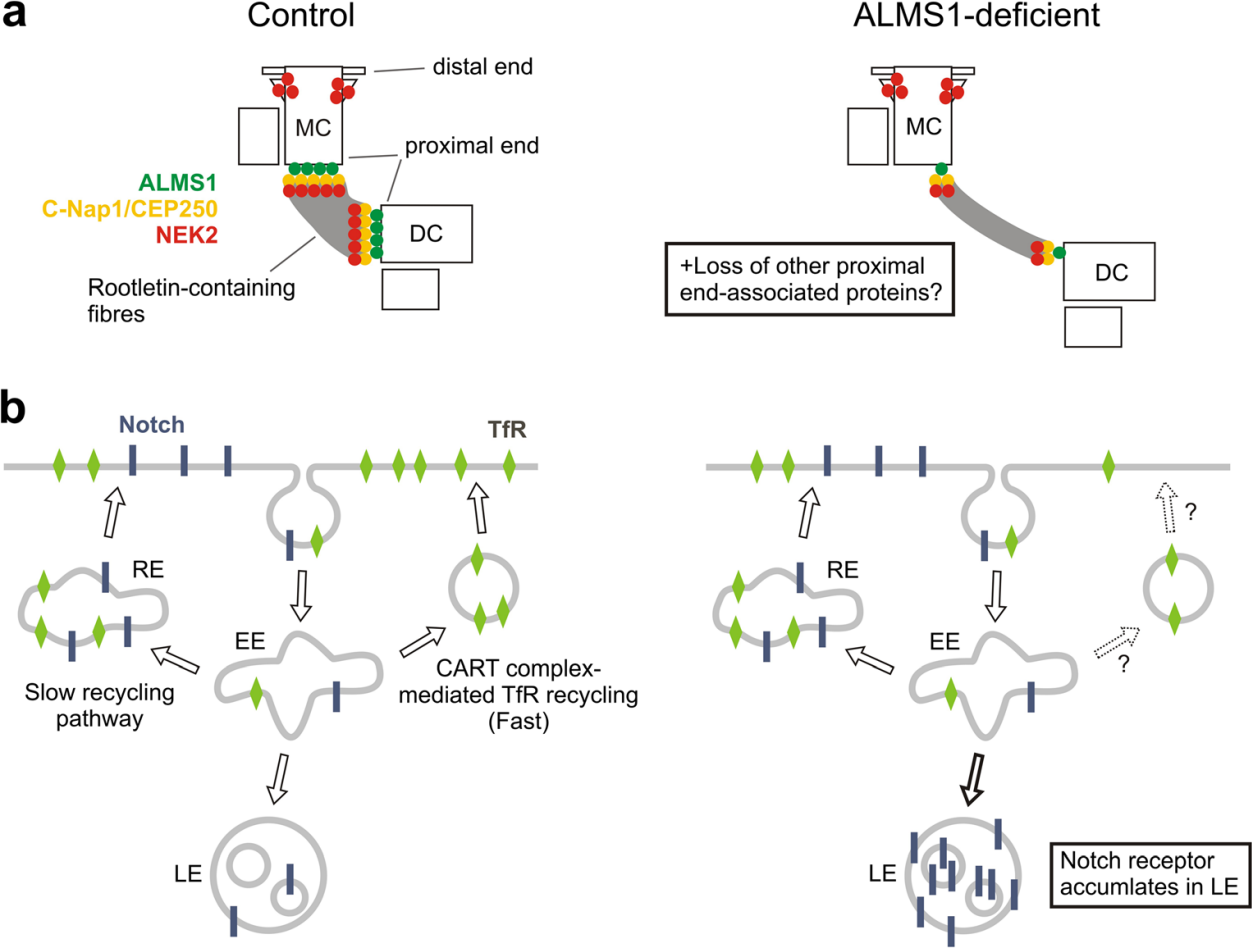
Potential involvement of ALMS1 in centrosome cohesion and endosomal trafficking. **a** ALMS1 has been implicated in anchoring the centrosome cohesion protein C-Nap1/CEP250 to centriole proximal ends [21], akin to CEP135 [186, 161]. Loss of CEP250 from these sites in ALMS1-depleted cells is accompanied by centrosome splitting [21]. The CEP250-associated kinase NEK2 is also lost, but not from the distal portion of the mother centriole (MC) [162]. The effect shown here on rootletin-containing fibres is inferred based on the rootletin-anchoring function of CEP250 [143, 144]. *DC* daughter centriole. **b** Endosomal trafficking of Notch and TfR (transferrin receptor) appears to be altered in ALMS1-deficient cells. Notch accumulates in late endosomes (LE) in ALMS1-depleted cells, potentially leading to increased signalling [74]. Clearance of Tf is delayed in fibroblasts derived from AS patients, suggesting impaired TfR recycling. Reported physical interactions between ALMS1 and CART complex components led to the suggestion that fast recycling of TfR may be impaired [76], indicated here by dashed arrows/question marks. *EE* early endosome, *RE* recycling endosome

The specific localisation of ALMS1 to the proximal ends of centrioles [21] is unusual for a ciliopathy-associated protein. Filaments emanating from this zone make up the centrosome linker, a structure that helps to maintain the close pairing of mother and daughter centrioles, termed centrosome cohesion ([143, 144]; Fig. 2). RNAi data have implicated ALMS1 in the proximal end-recruitment of CEP250/C-Nap1 [21], a protein that anchors the centrosome linker and a related structure called the ciliary rootlet (Figs. 2 and 5a) [143–145]. Consistently, centrosome cohesion appears to be compromised in ALMS1-depleted cells ([21]; Fig. 5a).

There is some evidence that mutations in human *CEP250* lead to retinal dystrophy and SNHL [146–148], and CEP250 is known to interact with proteins implicated in rhodopsin trafficking (NEK2 and NINL) [149–152]. It is also worth noting that human Tubby and the core PCP protein Dvl2 localise to the rootlet [153, 154]. CEP250 does not seem to be required for primary cilium formation or maintenance [59, 155–157], although a recent conflicting report suggests that ciliogenesis cannot proceed if centrosome cohesion is lost [158]. Another recent study showed that CEP250 contributes to retaining the cilium in a submerged state, affecting its capacity to sense external cues [159]. CEP250 appears to be crucial for the recruitment of several proteins to centriole proximal ends, including Girdin (Girders of actin filaments) and a group of proteins that associate also with the distal region of the mother centriole (NEK2, ninein, CEP170, p150glued and KIF2A) [159–162]. Thus, recruitment of these proteins to centriole proximal ends may depend indirectly on ALMS1, and there is evidence of this in the case of NEK2 ([162]; Fig. 5a).

In addition to CEP250 and NEK2, centrosomal levels of the PCM protein PCNT are modestly reduced in ALMS1-depleted hTERT-RPE1 cells [21]. Like ALMS1, PCNT has been implicated in ciliogenesis, maintenance of centrosome cohesion and adipogenesis [31, 163, 164]. Moreover, *PCNT* mutations lead to severe insulin resistance, dyslipidemia and diabetes in the context of microcephalic osteodysplastic primordial dwarfism type 2 (MOPDII) [31]. Centrosomal recruitment of PCNT is mediated in part by polyglutamate chains added post-translationally to centriolar tubulin [165], making it tempting to speculate that the N-terminal polyglutamate tract of ALMS1 could act in a similar fashion.

It is not hard to imagine ALMS1 performing a structural role such as tethering other proteins to the base of centrioles. Alternatively, it could influence centrosome composition indirectly. Recent data indicate that pericentrosomal levels of centriolar satellites, key regulators of centrosome composition, are enhanced in ALMS1-depleted cells [131]. However, it should be emphasised that the above findings are based on RNAi and await confirmation by rescue experiments and genetic studies.

### Actin cytoskeleton organisation

Immunofluorescence analysis of filamentous actin in AS patient fibroblasts revealed the presence of non-uniform and stunted filaments, in addition to stress fibres similar to those of control cells [76]. A separate electron microscopy-based analysis of AS fibroblasts revealed abnormal orientation of microfilaments (a.k.a. actin filaments) [75]. Also of note, these cells displayed a reduced ability to migrate in 3D culture, strong anchoring of cytoplasmic extensions, increased production of extracellular matrix (ECM), an extended cell cycle and resistance to chemically induced apoptosis [75]. It is not known how ALMS1 dysfunction leads to defects in F-actin organisation, although, as noted above, physical interactions with actin-binding/regulatory proteins have been reported [76, 113]. It remains to be investigated if ALMS1 is important for the recently discovered ability of centrosomes to nucleate actin filaments [25].

### Endosomal trafficking of transferrin and Notch

Impairments in the trafficking and recycling of endocytosed transferrin (Tf) have been noted in AS patient fibroblasts [76]. The transferrin receptor showed enhanced accumulation in the pericentrosomal region, while uptake of labelled transferrin was slightly impaired and its exit from the recycling compartment delayed compared to control fibroblasts. The positioning of the pericentrosomal endosomal recycling compartment, marked by Rab11, was unaffected. In the same study, an antibody raised to a C-terminal peptide of murine Alms1 was found to stain the contractile ring and cleavage furrow (sites to which the endosomal recycling machinery is recruited) as well as cytoplasmic ACTN1-immunopositive dense bodies, but not centrosomes, in Madin-Darby canine kidney (MDCK) cells. Based on these and additional data mentioned above, it was speculated that ALMS1 may be required for the cytoskeletal architecture that enables endosomal recycling, and/or function together with the CART complex to facilitate rapid endosome transport along actin filaments ([76]; Fig. 5b).

Endosomal trafficking of Notch, a receptor involved in multiple processes including cell fate determination during development, also appears to be disrupted in ALMS1-deficient cells, although in this case the recycling branch of the pathway was not significantly affected [74]. Specifically, Notch was reported to accumulate in late endosomes in ALMS1-depleted hTERT-RPE1 cells and a slight (1.2-fold) but statistically significant increase in expression of the Notch target gene *HES5* was detected in ALMS1-depleted HEK293 cells (Fig. 5b). Overactivation of Notch signalling could have wide-ranging impacts relevant to the pathogenesis of AS including extending the proliferative window of neonatal cardiomyocytes and exacerbating selective insulin resistance in the liver [166, 167]. Notch signalling has been reported to utilise the primary cilium [168]; however, ALMS1 does not appear to be required for localisation of NOTCH1 to the basal body or cilium [74]. Of note, depletion of BBS proteins similarly leads to accumulation of Notch in late endosomes but also impairs endocytic recycling and basal body/ciliary localisation of the receptor [74].

### GLUT4 trafficking and/or adipogenesis

Indications that ALMS1 influences endocytic recycling suggested that it could have a role in glucose transporter type 4 (GLUT4) trafficking [76]. Insulin-stimulated movement of GLUT4 from intracellular stores to the plasma membrane in fat and muscle cells plays a key role in maintaining whole-body glucose homeostasis [169]. Analysis of adipose tissue from *Alms1*^GT/GT^ mice revealed that the level of GLUT4 in the plasma membrane fraction was higher in the basal than the insulin-stimulated state, and that the overall level of GLUT4 was reduced compared to control [73]. Moreover, *Alms1*^GT/GT^ preadipocytes differentiated in vitro showed reduced insulin-stimulated GLUT4 translocation and glucose uptake compared to wild type cells [73]. Phosphorylation of Akt, the main insulin-regulated signal transducer for GLUT4 translocation, appeared to be unaffected in *Alms1*^GT/GT^ adipose tissue, suggesting that ALMS1 functions either downstream or independently of Akt [73]. The actin cytoskeletal defects observed in patient fibroblasts and the reported physical interaction between Alms1 and Actn4 [76] may provide clues to the underlying mechanism [73, 170]. However, as outlined below, there are conflicting data regarding the importance of Alms1 for insulin-stimulated glucose uptake (and, by implication, GLUT4 trafficking).

Earlier RNAi experiments using 3T3-L1 preadipocytes indicated that Alms1 is important for adipogenic differentiation [79], whereas preadipocytes from *Alms1*^GT/GT^ mice showed no such differentiation defect in vitro [73]. This discrepancy may reflect cell line specificity or a difference in the level of residual Alms1 function present, as suggested to explain the ability of patient fibroblasts and Alms1 mutant mice to assemble morphologically normal primary cilia [57]. Of note here, signalling pathways with positive (IGF-1R) and negative (Wnt and Hh) effects on adipogenesis utilise cilia [45, 95, 171], but whether ALMS1 deficiency affects ciliation during this process is not known.

Similar to the later analysis of *Alms1*^GT/GT^ mouse adipose tissue, Alms1 did not appear to be required for proximal insulin signalling in 3T3-L1 adipocytes [79]. However, neither did it seem to be required for insulin-stimulated glucose uptake, since the modest decreases observed in Alms1-depleted 3T3-L1 adipocytes could be accounted for by impaired differentiation [79]. This study suggests an alternative mechanism contributing to insulin resistance in AS. Namely, that a partial defect in adipogenesis would reduce the ability of adipose tissue to expand in situations of caloric excess, leading to the development of insulin resistance at other sites [79, 172]. However, since the severity of insulin resistance in AS often exceeds that in congenital generalised lipodystrophy, other factors are likely involved [79].

### Transcription

As noted earlier, ALMS1 has been identified as a potential binding partner of the RNAPII subunit RPB1 [77]. In the same study, punctate nuclear (and cytoplasmic) anti-ALMS1 staining was observed with two different antibodies in cryosections of HeLa cells and murine tissues, and immunolabeling of ALMS1 was found to block subsequent immunolabeling of RPB1, suggesting colocalisation (based on antibody steric hindrance). Supporting the biological relevance of these findings, ALMS1 knockdown reduced global transcription levels in HeLa cells. Thus, these data suggest that ALMS1 localises to the nucleus and is important for sustaining the transcriptional activity of RNAPII [77]. Deficiency of ALMS1 has been associated with altered gene expression profiles in other systems [75, 173, 174]; however, it is unclear if these effects are direct. Of note, the transcriptional program associated with ciliogenesis/confluency was unaffected in Alms1-depleted IMCD3 cells [57], suggesting that impaired transcription is unlikely to underlie the severe ciliary defects observed in these cells.

## Conclusions

Perhaps not surprisingly given the complexity of the AS phenotype, ALMS1 appears to have multiple functions. The photoreceptor phenotype of *Alms1*^GT/GT^ mice hints at involvement in the transition from vesicular to intraciliary transport, a process controlled in part by the small GTPase Rab8 and in which ciliopathy proteins including CC2D2A and BBS proteins have been implicated [61, 151, 175, 176]. Other studies suggest that ALMS1-deficiency leads to a wider impairment of vesicular transport, affecting Notch signalling, GLUT4-mediated glucose uptake and potentially many other processes. It remains unclear if the underlying cause of these transport deficits is disruption of the actin cytoskeleton (as observed in AS patient fibroblasts), of MTs or of specific trafficking steps. Following up on leads from protein-protein interaction data, recently significantly enriched by a human interactome study [113], may help to clarify this. Notably, perturbation of either the actin cytoskeleton or the endocytic recycling pathway can affect ciliogenesis and cilium length [114]. However, as discussed earlier, the importance of ALMS1 for cilium structure remains somewhat uncertain, due in part to the lack of obvious ciliary defects in patient-derived fibroblasts.

The application of proximity labelling has raised the intriguing possibility that ALMS1 interacts with centriole assembly factors, similarly to the ALMS motif-containing protein CEP295/KIAA1731. Although centriole-loss has not been observed in ALMS1-deficient cells, a subtler defect, which could affect both ciliary and extraciliary functions of the centrosome, remains a possibility. Loss of the centrosome cohesion protein CEP250/C-Nap1 from ALMS1-depleted centrioles could be a manifestation of such a defect. Indications that ALMS1 has roles in transcription [77] and possibly the DDR [89, 91] suggest additional areas for further research.

Immunofluorescence data placing ALMS1 at locations such as the nucleus and cleavage furrow support the idea that multiple isoforms of the protein exist. However, it is important to note that these localisation data have yet to be confirmed by, for example, parallel analysis of ALMS1-depleted cells. Further work is needed to explore the range of *ALMS1* transcripts produced by cells, their functional capacities and potential relevance to phenotypic variability in AS.

It remains plausible that primary cilium or basal body dysfunction contributes to many aspects of the AS phenotype including obesity, retinal dystrophy, hearing-loss, kidney dysfunction, neurological disturbances and fibrosis [48, 52, 78]. Moreover, data implicating pancreatic β cell primary cilia in the regulation of insulin secretion [177] raise the possibility that a ciliary defect contributes to the progressive failure of insulin secretion that occurs in AS and is the major factor in progression to diabetes [178]. Dysfunction of *motile* cilia could contribute to other clinical aspects of AS including respiratory problems, chronic otitis media in childhood and abnormal spinal curvature in adolescence [179, 180]. Although evidence that ALMS1-deficiency directly impacts motile cilia is lacking at present, upregulation of *ALMS1* expression during MCC differentiation is intriguing.

Whether a ciliary defect could impair cardiomyocyte cell cycle arrest, leading to infantile DCM/mitogenic cardiomyopathy, is uncertain. Signalling pathways linked to primary cilia in other contexts (e.g. Hippo, Wnt, IGF-1R, Notch) are among the many factors thought to regulate this arrest [167, 181]. However, mammalian cardiomyocytes appear to lose the ability to assemble a primary cilium shortly after birth, concomitant with loss of centrosome integrity and redistribution of centrosomal proteins to the nuclear periphery [182].

Clearly, much remains to be learned about this giant protein. The identification of bona fide interacting partners remains an important goal, as does, more straightforwardly, confirmation of extra-centrosomal sites of localisation. Structural data may reveal homologies not detectable at the sequence level, helping to reveal the functions of individual domains. The establishment of zebrafish models of AS [80, 173] will facilitate in vivo functional studies, the identification of genetic interactions and therapeutic testing/screening. However, the relatively poor evolutionary conservation of ALMS1 is a limitation of non-mammalian models. Gene editing and the generation of patient-derived induced pluripotent stem cells are likely to play important roles in resolving the functions of the human protein. While this article was in production, it was reported that Alms1 physically interacts with and promotes endocytosis of the renal Na-K-Cl cotransporter NKCC2 in the thick ascending limb (TAL) of the loop of Henle [187]. Alms1 knockout rats developed hypertension (a feature observed in 30% of AS patients [4]), suggested to be due at least in part to NKCC2 accumulation at the apical cell surface and higher TAL NaCl transport. Kidney tubule primary cilia were reported to be of normal length.
